# 基于紫外诱导可见发光成像和热裂解气相色谱-质谱的铁质文物保护修复材料鉴别

**DOI:** 10.3724/SP.J.1123.2024.02005

**Published:** 2024-10-08

**Authors:** Qin YANG, Li DING, Zhaohui LI, Ran ZHANG, Yue WEI, Ying CHEN

**Affiliations:** 1.中国国家博物馆, 金属文物保护国家文物局重点科研基地(中国国家博物馆), 北京 100006; 1. National Museum of China, Key Scientific Research Base of Metal Conservation (National Museum of China), National Cultural Heritage Administration, Beijing 100006, China; 2.甘肃省博物馆, 甘肃 兰州 730050; 2. Gansu Provincial Museum, Lanzhou 730050, China; 3.昭通市博物馆, 云南 昭通 657099; 3. Zhaotong Municipal Museum, Zhaotong 657099, China

**Keywords:** 紫外诱导可见发光成像, 热裂解气相色谱-质谱, 保护修复材料, 铁质文物, ultraviolet-induced visible luminescence imaging (UVL), pyrolysis-gas chromatography/mass spectrometry (Py-GC/MS), conservation and restoration materials, iron relics

## Abstract

在制订文物保护方案前,首先需先了解其保存现状和历史保护修复情况,过往修复所用的材料成分和保存状态是现状调查的重要内容。由于早期部分文物保护工作缺乏详细的档案记录,采用科学检测方法识别保护修复材料至关重要。本研究运用紫外诱导可见发光成像(UVL)与热裂解气相色谱-质谱(Py-GC/MS)技术,对收藏于甘肃省博物馆、昭通市博物馆、中国国家博物馆的5件铁质文物的历史保护修复材料进行了成分和空间分布的调查。结果显示,铁权20791和铁锛头2335使用了添加松香树脂的熟桐油作为封护材料;铁斧2334的封护材料由两层组成,下层为熟桐油,上层为虫胶;铁剑D0008使用了石蜡封护;铁剑450保护过程中应用了多种材料,包括双酚A型环氧树脂、虫胶、聚苯乙烯等。研究证实UVL与Py-GC/MS结合使用是分析历史保护修复材料的有效方法,点分析和成像技术的结合为取样策略的制定提供了依据,确保了样本的代表性,且减少了取样数量和对文物的潜在损害。研究结果为文物档案补充了重要信息,为文物修复材料效果评估、失效保护材料去除以及后续保护方案制订提供了科学依据。

## Abstract (en)

Understanding the previous protection and restoration efforts and the current state of cultural relics is essential before compiling a conservation and restoration plan. The lack of detailed archival records for some early conservation operations, the identification of restoration materials necessitates the use of scientific analytical methods. In this study, the composition and spatial distribution of historical restoration materials on five iron relics were investigated through pyrolysis-gas chromatography/mass spectrometry (Py-GC/MS) and ultraviolet-induced visible luminescence imaging (UVL). The relics studied were iron weight 20791, iron adze head 2335, and iron axe 2334 from the Gansu Provincial Museum, iron sword D0008 from the Zhaotong Municipal Museum, and iron sword 450 from the National Museum of China. All five relics had undergone restoration without accompanying archival records. UVL revealed the distribution of various conservation materials. Notably, two distinct layers of the conservation material were observed on iron axe 2334. Differences in the fluorescence color and intensity of iron sword 450 provided information regarding the sampling strategy. The samples were collected under ultraviolet light emitting diode illumination to ensure representativeness and minimize damage to the relics. Through Py-GC/MS, the coating materials for iron weight 20791 and iron adze head 2335 were identified as boiled tung oil mixed with rosin resin. Iron axis 2334 had a two-layer coating: a base layer of boiled tung oil and a top layer of shellac. The coating material for iron sword D0008 was determined to be paraffin wax. The protective layer of iron sword 450 included multiple materials, including shellac, polystyrene, and bisphenol-A-type epoxy resin. This study confirms that UVL combined with Py-GC/MS serves as an effective technique for analyzing historical restoration materials. UVL guided the selection of representative samples for Py-GC/MS, reducing the time and amount of sampling required and minimizing further damage to the relics. This research provides valuable data for the restoration archives of five iron artifacts, offering a scientific basis for conservators to evaluate conservation methods, devise future conservation strategies, and exclude ineffective conservation materials.

铁在人类社会和文明发展中起着重要的作用。中国早在商代就开始利用陨铁,至迟在春秋时期发明了冶铁技术,铁制品逐步应用到生产生活的方方面面。铁的化学性质比较活泼,容易被腐蚀,因此与铜、金、银等其他金属文物相比,铁质文物的锈蚀情况通常更加严重,其保护也更为困难。铁质文物的保护流程包括分析检测、清洗除锈、脱盐、加固、粘接、做旧、缓蚀、封护、环境控制等,保护过程中所使用的保护材料对于文物的长期保存至关重要。虽然文物保护材料的选择考虑了材料的耐久性,但是随着时间流逝,部分保护材料仍出现老化变色、失去保护效果、引起新的病害等情况。因此,保护材料的表征和失效保护材料的去除逐渐引起文物保护工作者的重视,目前相关的研究主要集中于壁画、油画保护材料的鉴定和清除^[1⇓⇓-4]^。

应用于铁质文物的保护材料大部分是天然和合成高分子材料,例如桐油、蜡、丙烯酸树脂、环氧树脂、氟碳树脂等^[5,6]^。早期使用的文物保护材料以天然材料为主,老一辈文物保护工作者逐渐将合成高分子材料引入文物保护工作之中。虽然,现今文物修复档案要求详细记录修复中使用的材料、工艺、步骤及操作条件,遗憾的是,在早期文物保护修复过程中,常存在文物修复信息无记录、记录不全或缺失的情况,这对文物状态的评估和再处理造成了困难。铁质文物历史保护修复材料的鉴定是其老化程度评价、失效评估的前提,也为选择“失效”保护材料去除方法、制订合理的保护方案提供科学的数据支撑。其次,铁质文物保护材料在文物表面的分布形态,不但反映了历史上文物保护工作者的修复策略与保护措施,还直观地展示了当前这些材料在文物表面附着的实际状况。早期保护修复材料的识别除可以通过对修复师的访谈调查外,现代科学技术包括红外光谱、拉曼光谱、气相色谱-质谱等已经被用于识别文化遗产保护领域使用的天然和合成聚合物^[7]^。本研究建立了基于紫外诱导可见发光成像(ultraviolet-induced visible luminescence imaging, UVL)和热裂解气相色谱-质谱技术(pyrolysis-gas chromatography/mass spectrometry, Py-GC/MS)的铁质文物历史保护修复材料鉴定和评估方法。宽带多光谱成像,尤其是UVL是区分文物原始材料和历史修复过程人为添加的保护材料的有效方法^[8,9]^,该方法具有无损便携、低成本、操作简单的优点,广泛应用于文物修复前后的状态调查和记录。Py-GC/MS是高分子材料鉴定和组成分析的有效方法^[10,11]^,所需样品量少,样品前处理简单,灵敏度高,信息量大。热辅助水解甲基化(thermally assisted hydrolysis-methylation, THM)-Py-GC/MS通过在样品中添加甲基化试剂如四甲基氢氧化铵(tetramethylammonium hydroxide, TMAH),在裂解器的高温辅助下与样品发生反应,使裂解产物中含有羧基、羟基等极性基团组分转变成相应的甲基化产物,降低其极性后再采用GC/MS分离和分析。两种方法的结合为文物历史保护修复材料的成分鉴定和分布调查提供了高效的方法。本研究通过UVL图像指导下的取样点选取和分析,对5件铁质文物的历史修复材料种类和分布进行了表征,为其保护效果的评估和保护方案的制订提供了科学依据。

## 1 实验部分

### 1.1 仪器与试剂

UVL光源1:U1c匀光紫外发光二极管(LED)手电(JAXMAN,中国),灯珠(Nichia,日本)功率6 W,峰值波长368 nm,配有厚度为2 mm的ZWB2滤光片,滤光片用于减少光源光谱中的可见光部分。该光源用于甘肃博物馆藏3件铁器的UVL图像拍摄,使用时光源与文物距离约50 cm。

UVL光源2: S3065-3K条形紫外LED灯(盐城市诺信电子设备有限公司),灯珠功率27 W,峰值波长为368 nm,配有厚度为2 mm的ZWB2滤光片。该光源用于中国国家博物馆藏铁剑的UVL图像拍摄,使用时光源与文物距离约1 m。

相机:D850单镜头反光相机,4575万有效像素,搭配AF-S Micro NIKKOR 60 mm f2.8G ED微距定焦镜头(Nikon,日本)。

EGA/PY-3030D微炉式裂解仪(Frontier Lab公司); 8860/5977B气相色谱-质谱仪(Agilent公司)。

25% TMAH甲醇溶液(麦克林试剂公司),切片石蜡(熔点58~60 ℃,阿拉丁试剂公司),虫胶(云南永德松桦林化制品有限责任公司)。

### 1.2 文物及参考样品

5件铁质文物的基本信息和保存现状如下:铁权,编号20791,收藏于甘肃省博物馆,汉代,三级文物,文物材质铁,通高6.6 cm,直径4.2 cm,整体保存状况较好,目视观察发现表面有明显光泽,疑似有封护层。铁锛头,编号2335,收藏于甘肃省博物馆,汉代,三级文物,文物材质铁,长10 cm,最宽8 cm,厚2 cm。铁斧,编号2334,收藏于甘肃省博物馆,汉代,三级文物,文物材质铁,文物长10.2 cm,宽6.2 cm,最厚3.7 cm。铁剑,文物编号D0008,通长118 cm,最宽3.4 cm,厚0.7 cm,藏于昭通市博物馆,据保管人员回忆,在出土后使用蜡类材料封护过。铁剑,编号450,藏于中国国家博物馆,汉代,保存状态较好。据修复师回忆,该铁剑于20世纪70年代末进行过修复,无档案记录,当时对铁器文物一般进行清洗、除锈、脱盐、粘接和封护^[12]^。

### 1.3 样品采集

通过UVL图像荧光颜色和强度初步判定修复材料的分布和异同,指导取样位置的选择。为了尽可能减少对文物原保护材料的损伤,在手持式紫外灯照射下,用手术刀沿着边缘或损坏的区域刮取少量样品,样品通过Py-GC/MS鉴定其成分。

### 1.4 Py-GC/MS分析条件

天然聚合物(方法1) 将约0.1 mg样品置于热裂解样品杯中,加入3 μL 25% TMAH甲醇溶液,随后送入热裂解仪石英裂解管。裂解温度550 ℃,裂解仪/GC接口温度320 ℃。GC条件:Ultra ALLOY^+^-5色谱柱(30 m×0.25 mm×0.25 μm,固定相为5%苯基-95%甲基聚硅氧烷,Frontier Lab公司);分流/不分流进样口温度320 ℃,柱箱升温程序为初始温度40 ℃,保持2 min,以6 ℃/min的速率升温至320 ℃,保持9 min。载气为高纯氦气,恒流模式,色谱柱流量1 mL/min,分流比根据样品量设置为50∶1~5∶1。质谱扫描参数:电子轰击(EI)离子源,离子源电压为70 eV,温度为230 ℃,四极杆温度为150 ℃,传输线温度为320 ℃。扫描范围*m/z* 33~600,溶剂延迟3 min。

合成高分子材料(方法2) 样品中不添加甲基化试剂。裂解仪裂解温度600 ℃,裂解仪/GC接口温度320 ℃。GC条件:分流/不分流进样口温度320 ℃,柱箱升温程序为柱箱初始温度40 ℃,保持2 min,以20 ℃/min的速率升温至320 ℃,并保持13 min。质谱扫描参数:扫描范围*m/z* 29~600,溶剂延迟1 min,其他参数同天然聚合物的MS条件。

定性 采集的数据利用Agilent MassHunter未知物分析软件处理,所得到的质谱图与NIST2017谱库进行匹配,通过正构烷烃标准样品计算各物质的保留指数(RI),以匹配因子阈值80、保留指数±50筛选谱库匹配的结果对化合物进行定性。样品中加入25% TMAH甲醇溶液在线甲基化时,识别的酸、醇等为甲基化产物。

## 2 结果和讨论

### 2.1 UVL结果

铁权20791的可见反射成像(visible-reflected imaging, VIS)和UVL图像见图1,铁权表面覆盖一层封护材料,其在UVL图像中呈现黄绿色。铁器锈蚀产物在紫外光激发下无荧光,因此在UVL图像中呈黑色,与封护材料形成强烈的反差,有助于识别封护层的分布。铁权封护层基本完整,中部和悬钮处有3处锈蚀剥落形成浅坑,露出黄色和深棕色锈蚀。另外,存在4处裂隙,裂隙区有继续发生锈蚀剥离的风险,UVL结果揭示铁权表面的封护层在多个区域呈现点状脱落,这些细微变化在可见光照射下并不明显。在封护层破损边缘取样,编号TQ-1(图1)。

**图1:**
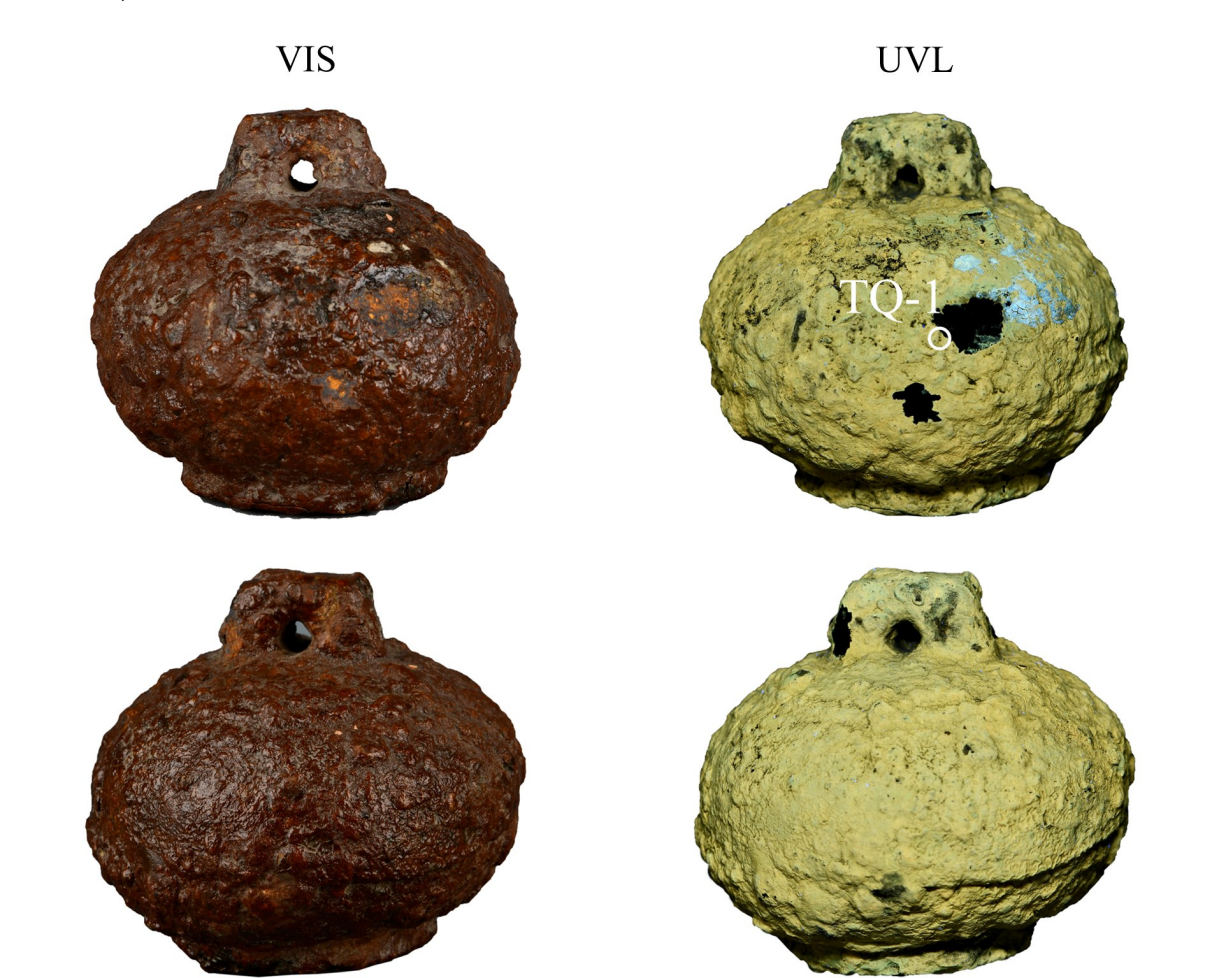
铁权20791的VIS和UVL图像(圆圈标注为 取样位置)

铁锛头2335的VIS和UVL图像见图2,成像结果表明其表层存在封护层,封护材料的荧光颜色和强度与铁权20791的封护材料相似。铁锛头2335保存状况较差,表层已出现大量片状锈蚀剥落,露出下层黄色、红褐色锈蚀产物。取残片表层封护材料样品,编号TBT-1。

**图2:**
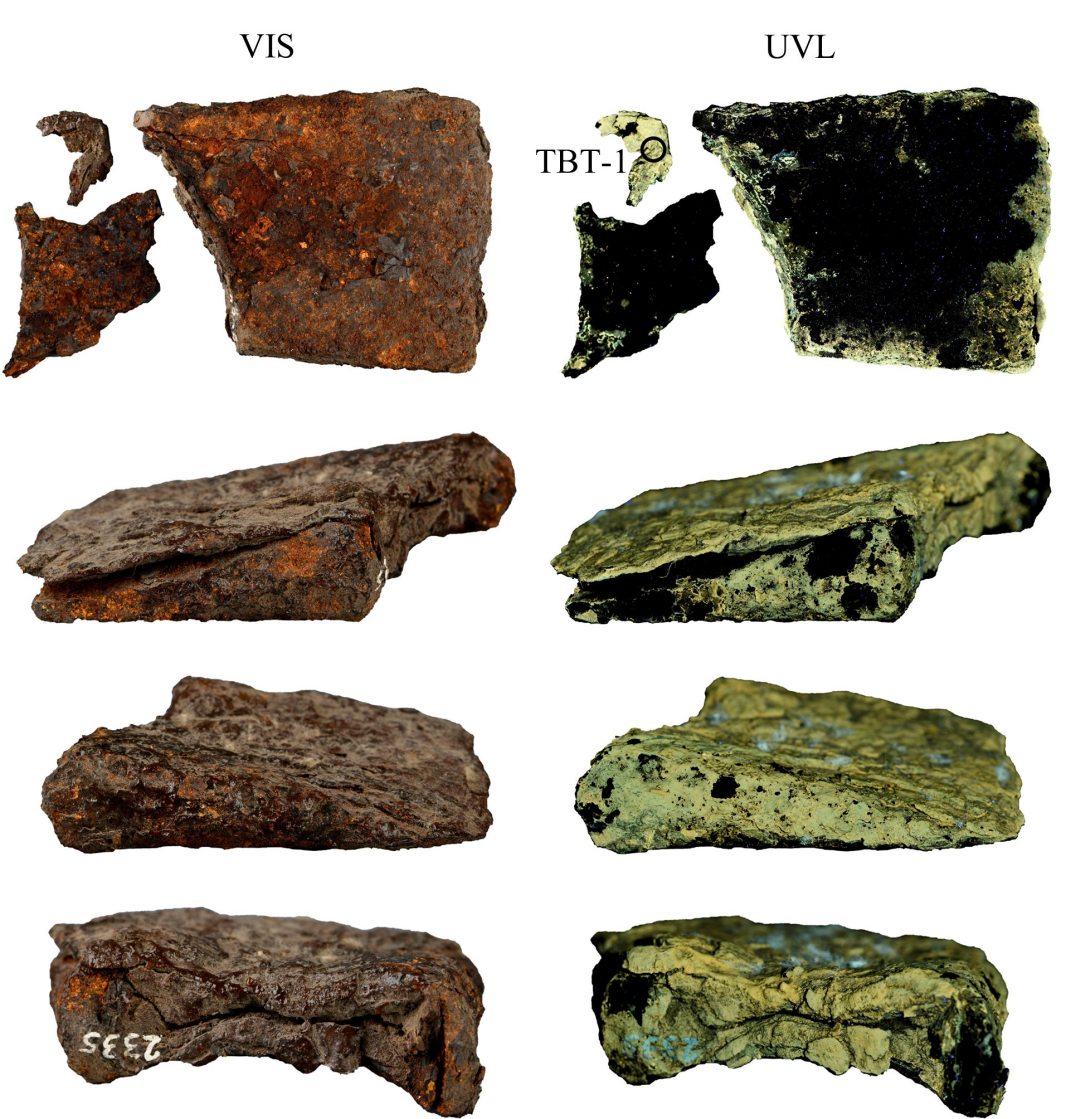
铁锛头2335的VIS和UVL图像 (圆圈标注为取样位置)

铁斧2334的VIS和UVL图像见图3。铁斧保存现状较差,外侧封护材料已经基本随锈蚀层剥落,掉落大小不一的片状、颗粒状锈蚀,剥落的片状表层为棕色,表层有光泽,UVL图像显示铁斧2334表层存在荧光橙色的封护层。銎口内侧壁封护层较为完好,UVL图像显示,橙色封护材料之下可见黄绿色荧光层,初步判断铁斧存在两层不同的封护材料,在紫外光照射下进行分层取样,以便对两层封护材料分别检测分析,取样位置和编号如图3所示。

**图3:**
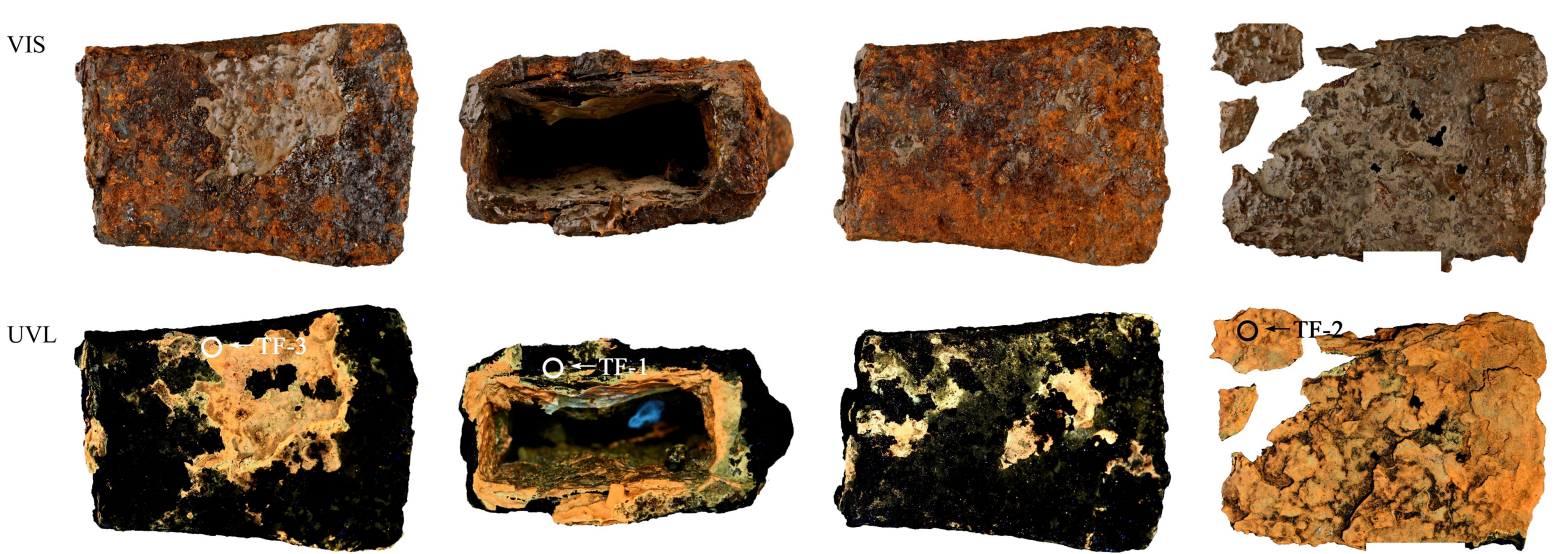
铁斧2334的VIS和UVL图像(圆圈标注为取样位置)

铁剑450整体保存状况良好,未见锈蚀剥落。通过VIS和UVL图像分析,观察到5种不同的荧光现象。为进一步研究,采集了5组10个样品,取样位置和编号见图4。这些样品包括荧光为天蓝色(GBTJ-1、GBTJ-2)、蓝色(GBTJ-3、GBTJ-4)、亮橙色(GBTJ-7、GBTJ-8)、暗橙色(GBTJ-9、GBTJ-10)以及无荧光有光泽(GBTJ-5、GBTJ-6)的区域。剑身中部荧光亮橙色材料贯穿剑身两侧,识别为粘接材料;荧光暗橙色材料覆盖于其他材料上,为随色作旧材料;荧光天蓝色材料主要分布在铁剑刃部,为补配材料;荧光蓝色材料主要位于剑柄附近的裂缝处,可能用于渗透加固或粘接;在可见光下,剑尖部位显示出光泽,推测在铁剑修复过程中使用了封护剂进行整体封护。

**图4:**
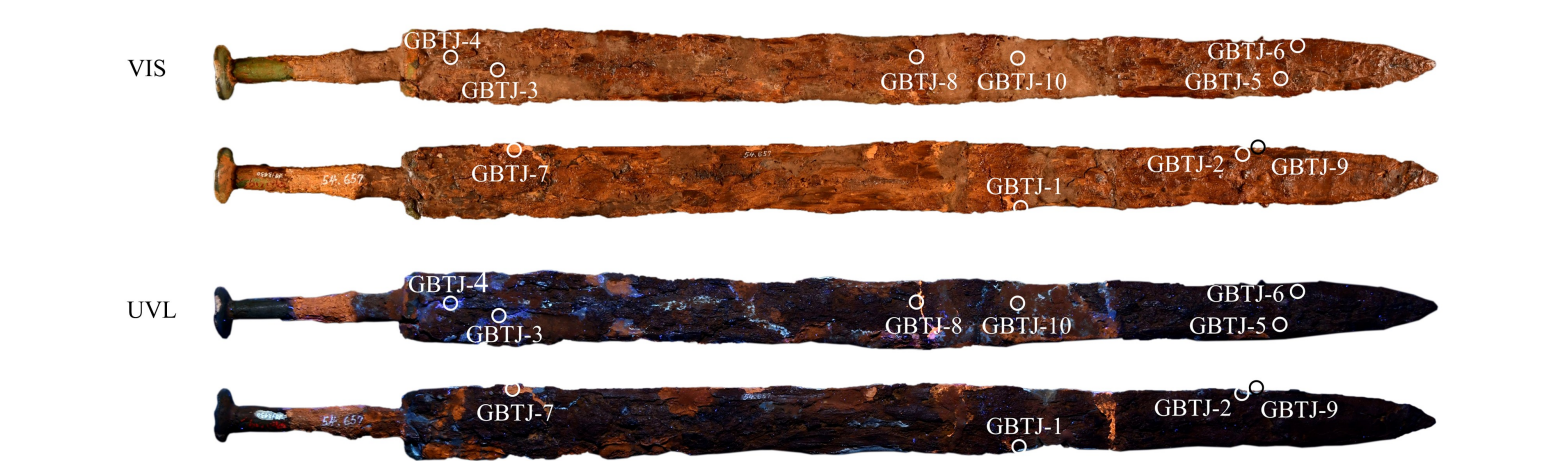
铁剑450的VIS和UVL图像(圆圈标注为取样位置)

### 2.2 Py-GC/MS分析结果

#### 2.2.1 甘肃省博物馆藏3件铁器历史封护材料

编号为TQ-1、TBT-1、TF-1(黄绿色荧光)以及TF-2、TF-3(橙色荧光)的样品和参考品的THM-Py-GC/MS总离子流色谱图如图5所示。

**图5:**
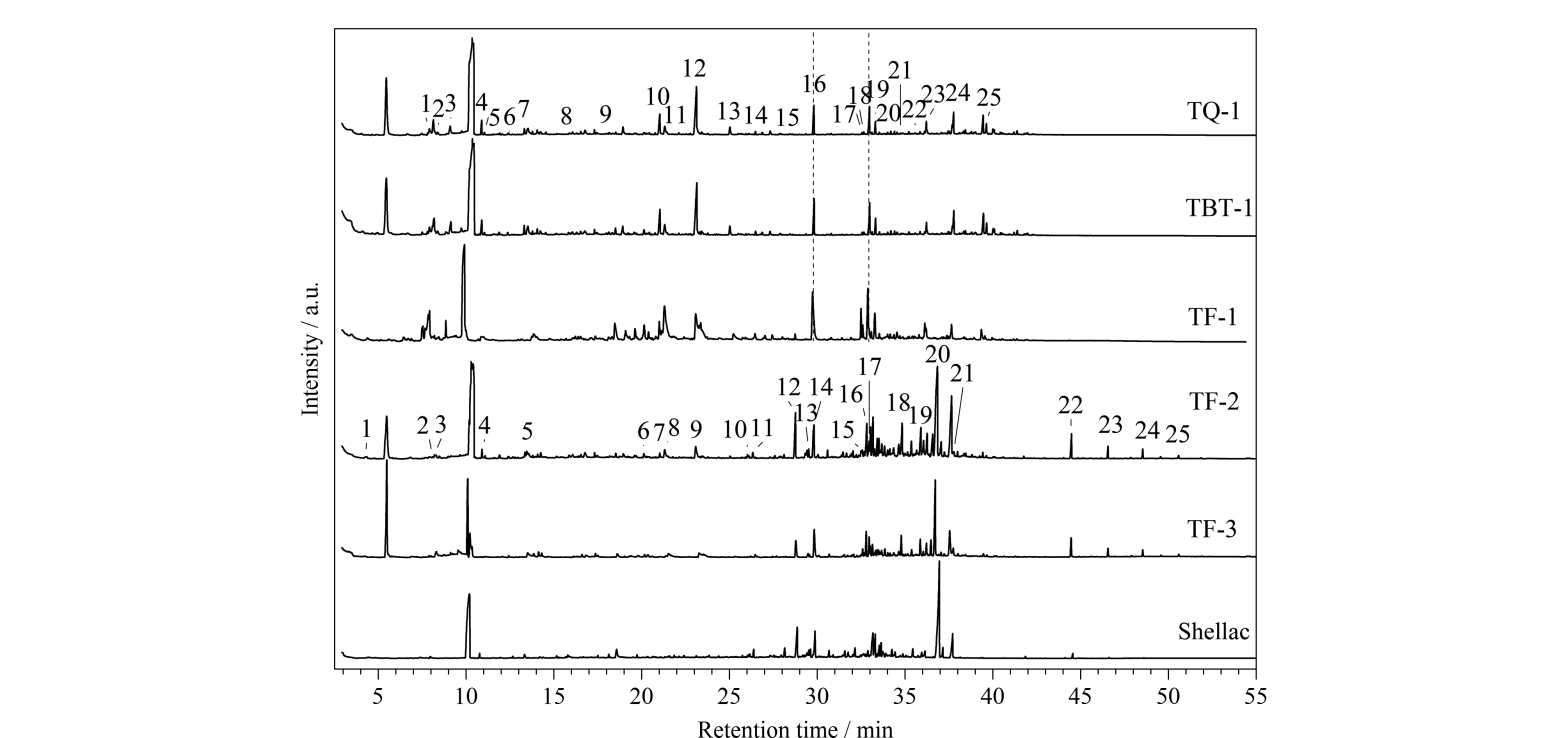
样品和虫胶样品的THM-Py-GC/MS总离子流图

TQ-1、TBT-1、TF-1 3个样品的THM-Py-GC/MS谱图基本相同,样品TQ-1的峰识别结果见表1。样品中检测到大量干性油的典型热裂解产物,如软脂酸、硬脂酸、庚酸等一元脂肪酸以及碳原子数为8~12的二元脂肪酸(以其甲酯、二甲酯形式存在),其中壬二酸含量最高,表明样品为干性油。根据文献[13,14]报道,可通过计算软脂酸和硬脂酸的峰面积比例(P/S)鉴定干性油种类,TQ-1样品的P/S值为1.12,与桐油的P/S区间相符。此外,通过提取*m/z* 105的色谱峰,在样品中识别到9-(邻丙基苯基)壬酸甲酯(保留时间为33.33 min,下同),质谱图和化学结构式见图6。该产物属于烷基苯基链烷酸酯类(alkylphenyl alkanoates, APAs),是桐油酸的3个共轭双键在加热至高温时环化产生,为熟桐油的特征热解产物^[15]^,进一步确认样品为熟桐油。样品中还检测到少量脱氢枞酸甲酯、7-甲氧基四脱氢枞酸甲酯等松科树脂生物标志物^[16]^,根据《营造法式》记载,传统桐油熬制一般会添加一定比例的土籽、樟丹等催干剂和松香等树脂^[17]^,推测熬制熟桐油时加入了少量松香。

**表1:** 样品TQ-1的THM-Py-GC/MS分析结果

Peak No.	t_R_/min	Compound	Match score	Formula	RI-RI(lib)^*^
1	8.06	propane, 1,2,3-trimethoxy-	97	C_6_H_14_O_3_	-1
2	8.51	hexanoic acid, methyl ester	81	C_7_H_14_O_2_	8
3	9.23	2,3-dimethoxypropan-1-ol	95	C_5_H_12_O_3_	-40
4	11.01	6-heptenoic acid, methyl ester	98	C_8_H_14_O_2_	-37
5	11.17	heptanoic acid, methyl ester	91	C_8_H_16_O_2_	6
6	12.17	2-hydroxyisocaproic acid, methyl ether, methyl ester	82	C_8_H_16_O_3_	-42
7	13.43	3-octenoic acid, methyl ester, (Z)-	89	C_9_H_16_O_2_	-8
8	15.97	8-nonenoic acid, methyl ester	83	C_10_H_18_O_2_	10
9	18.25	4-decenoic acid, methyl ester	80	C_11_H_20_O_2_	-18
10	21.10	octanedioic acid, dimethyl ester	98	C_10_H_18_O_4_	10
11	21.39	dimethyl phthalate	93	C_10_H_10_O_4_	2
12	23.19	nonanedioic acid, dimethyl ester	99	C_11_H_20_O_4_	7
13	25.09	decanedioic acid, dimethyl ester	93	C_12_H_22_O_4_	11
14	26.92	undecanedioic acid, dimethyl ester	88	C_13_H_24_O_4_	12
15	28.66	dodecanedioic acid, dimethyl ester	80	C_14_H_26_O_4_	16
16	29.84	hexadecanoic acid, methyl ester	98	C_17_H_34_O_2_	20
17	32.57	10-octadecenoic acid, methyl ester	92	C_19_H_36_O_2_	26
18	32.67	9-octadecenoic acid, methyl ester, (E)-	93	C_19_H_36_O_2_	25
19	32.99	methyl stearate	95	C_19_H_38_O_2_	22
20	33.33	nonanoic acid, 9-(o-propylphenyl)-, methyl ester	82	C_19_H_30_O_2_	39
21	34.77	sandaracopimaric acid methyl ester	80	C_21_H_32_O_2_	25
22	35.86	methyl 18-methylnonadecanoate	85	C_21_H_42_O_2_	-12
23	36.22	methyl dehydroabietate	92	C_21_H_30_O_2_	7
24	37.76	tetradehydroabietic acid, 7-methoxy-, methyl ester	87	C_22_H_30_O_3_	-25
25	39.62	7,15-dimethoxytetradehydroabietic acid, methyl ester	80	C_23_H_32_O_4_	-15

*RI-RI(lib) is the retention index variation.

**图6:**
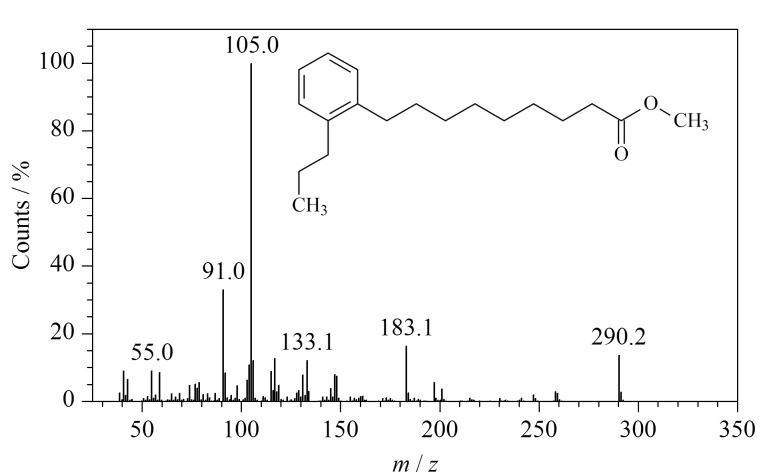
9-(邻丙基苯基)壬酸甲酯(*t*_R_=33.33 min) 的质谱图和化学结构式

样品TF-2和TF-3的总离子流图基本相同,TF-2的THM-Py-GC/MS裂解产物分析结果见表2。在样品裂解产物中识别出了紫铆醇酸甲酯甲醚(28.80 min)、紫胶壳脑酸二甲酯甲醚(32.844 min)、壳脑酸二甲酯二甲醚(34.85 min)、紫胶桐酸甲酯三甲基醚(36.85 min)等虫胶的生物标记物,表明该封护材料为虫胶树脂。部分目标化合物的质谱图和化学结构式如图7所示。

**表2:** 样品TF-2的THM-Py-GC/MS分析结果

Peak No.	t_R_/min	Compound	Match score	Formula	RI-RI(lib)
1	4.48	toluene	88	C_7_H_8_	11
2	8.23	propane, 1,2,3-trimethoxy-	81	C_6_H_14_O_3_	-8
3	8.34	5-hexenoic acid, methyl ester	88	C_7_H_12_O_2_	-36
4	11.03	6-heptenoic acid, methyl ester	97	C_8_H_14_O_2_	-37
5	13.47	3-octenoic acid, methyl ester, (Z)-	81	C_9_H_16_O_2_	-10
6	20.20	longifolene	94	C_15_H_24_	9
7	21.11	octanedioic acid, dimethyl ester	96	C_10_H_18_O_4_	9
8	21.39	dimethyl phthalate	96	C_10_H_10_O_4_	2
9	23.14	nonanedioic acid, dimethyl ester	94	C_11_H_20_O_4_	10
10	26.08	methyl myristoleate	92	C_15_H_28_O_2_	25
11	26.38	methyl tetradecanoate	94	C_15_H_30_O_2_	18
12	28.80	butolic acid, methyl ester, methyl ether	95	C_16_H_32_O_3_	13
13	29.56	9-hexadecenoic acid, methyl ester, (Z)-	95	C_17_H_32_O_2_	9
14	29.85	hexadecanoic acid, methyl ester	97	C_17_H_34_O_2_	19
15	32.69	11-octadecenoic acid, methyl ester	82	C_19_H_36_O_2_	29
16	32.84	laccishellolic acid, dimethyl ester, methyl ether	97	C_18_H_26_O_5_	18
17	32.97	methyl stearate	95	C_19_H_38_O_2_	23
18	34.85	shellolic acid, dimethyl ester, dimethyl ether	95	C_19_H_28_O_6_	14
19	36.26	methyl dehydroabietate	94	C_21_H_30_O_2_	3
20	36.85	aleuritic acid, methyl ester, trimethyl ether	95	C_20_H_40_O_5_	5
21	37.78	tetradehydroabietic acid, 7-methoxy-, methyl ester	83	C_22_H_30_O_3_	-26
22	44.44	1-methoxyoctacosane	96	C_29_H_60_O	28
23	46.52	methyl triacontyl ether	94	C_31_H_64_O	37
24	48.48	dotriacontyl methyl ether	93	C_33_H_68_O	37
25	50.52	methyl tetratriacontyl ether	84	C_35_H_72_O	41

**图7:**
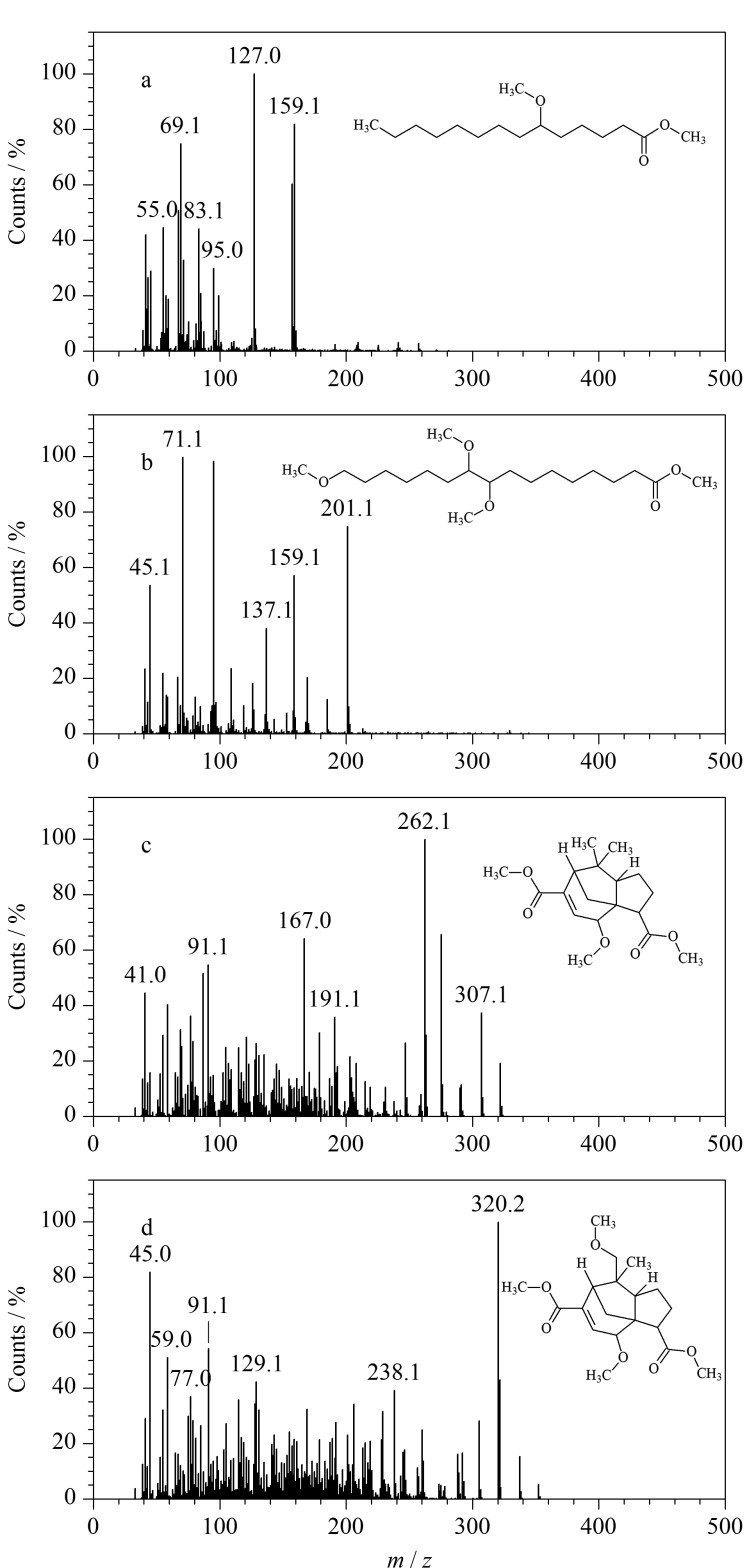
样品TF-2部分化合物的质谱图和化学结构式

虫胶又叫紫胶、紫虫胶、赤胶、紫草茸等,是由紫胶虫分泌的天然物质,主要由树脂、蜡、色素组成,其中树脂含量占65%~80%,紫胶树脂主要是由羟基脂肪酸和羟基倍半萜烯酸构成的聚酯混合物^[18]^。虫胶是一种重要的化工原料,用途广泛,在青铜器、铁器保护中,虫胶通常溶于酒精(称为“漆皮酒精”)中使用,用于粘接、调和颜料作色等,过去也用于艺术品和乐器的封护涂层。在紫外光照射下,虫胶能发出橙色荧光,可用于初步判断其存在与否。

Py-GC/MS分析表明,铁权20791、铁锛头2335的封护材料为添加了松香的熟桐油。铁斧2334表层封护材料分为两层,底层为熟桐油,表层为虫胶,表明其至少经历过两次修复过程。较早的修复采用了和铁权、铁锛头同样的材料,可能为同批次完成保护处理,后续在原封护层上直接进行了第二次封护。桐油和虫胶树脂作为传统的铁质文物封护材料,在当前的保护实践中已较少使用。本研究案例中的成功鉴定,揭示了其在历史上的应用。3件铁器保存状况不佳,封护材料已经失效,未能提供有效的保护。该批样品的大幅面X射线荧光成像结果显示样品中含有大量的Cl元素(此部分数据由项目组其他人员采集,待发表),推测在封护前未进行彻底的脱盐,其保存状态较差可能和脱盐不充分或者未脱盐有关。

#### 2.2.2 铁剑D0008历史封护材料

在UV LED光源的照射下,对铁剑D0008的保护材料进行了观察,铁剑呈现出浅蓝色荧光。为避免破坏封护层的完整性,选择荧光强度较大的区域,用手术刀谨慎地刮取表层材料,所得样品为粉末状,样品编号TJ-1。样品TJ-1的Py-GC/MS结果(图8)显示其主要由碳原子数分布在20~37(C_20_~C_37_)的直链烷烃组成,各烷烃峰面积近似正态分布,与熔点为58~60 ℃的切片石蜡色谱峰一致,该封护材料鉴定为石蜡。蜡类封护材料是铁质文物最常用的封护材料之一,用于铁质文物保护的蜡类材料有石蜡、微晶石蜡、虫白蜡、棕榈蜡等。石蜡熔点低,耐温性差,高熔点的微晶石蜡目前使用更加广泛^[19]^。蜡类封护材料防腐蚀性和耐候性能比清漆类差,预期寿命短^[20]^。因此,以石蜡作为封护材料的铁剑,需定期监测封护层的保护效果并适时进行养护。

**图8:**
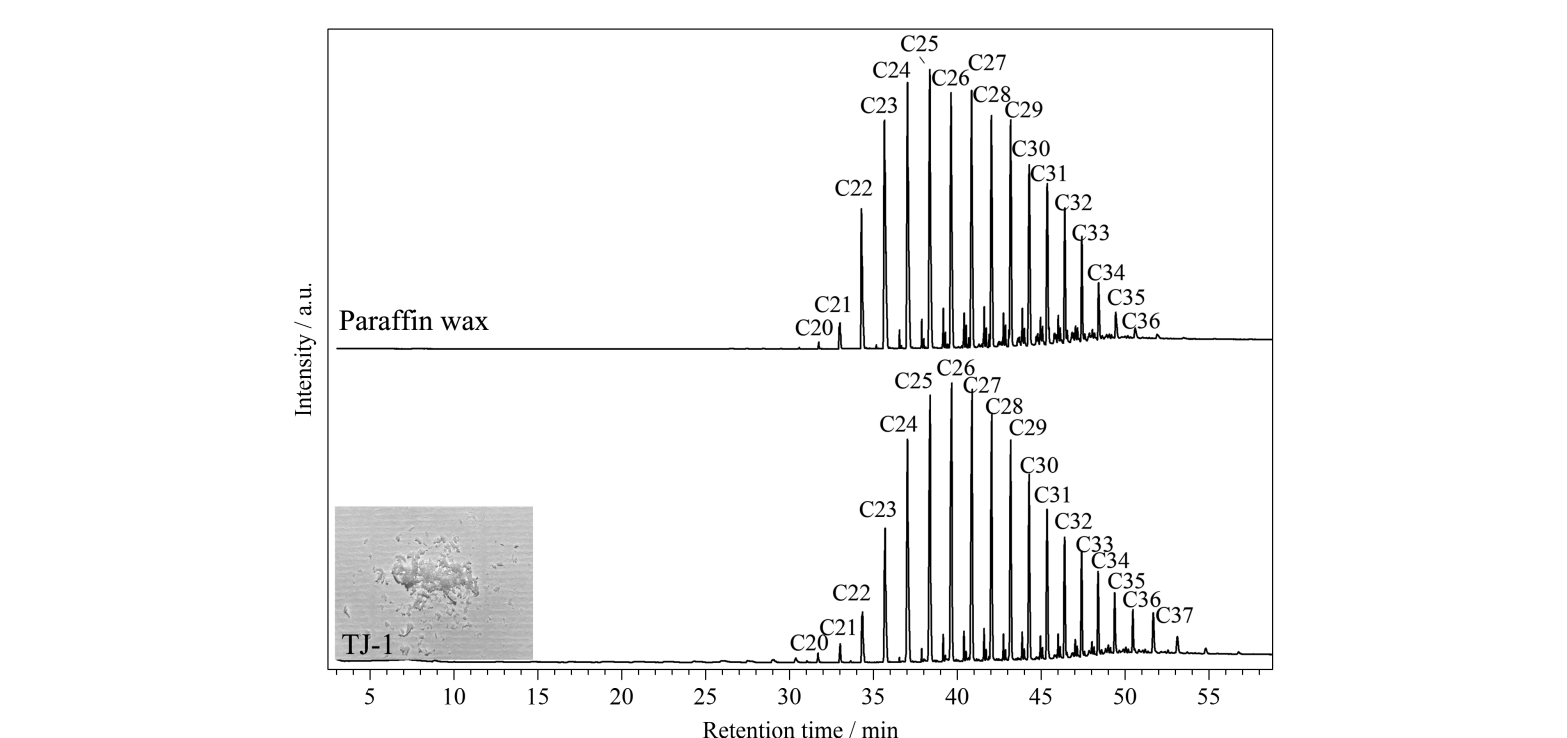
样品TJ-1封护材料和切片石蜡的Py-GC/MS总离子流图

#### 2.2.3 铁剑450历史修复材料

铁剑450样品GBTJ-1~6的Py-GC/MS结果见图9。GBTJ-1、GBTJ-2主要裂解产物为双酚A、苯酚、对异丙基酚、对烯丙基酚等,表明样品为双酚A型环氧胶黏剂。GBTJ-3、GBTJ-4主要裂解产物为苯乙烯单体、二聚体和少量三聚体,其他热解产物包括α-甲基苯乙烯、1,2-二苯基乙烷、1,2-二苯基丙烷、2,5-二苯基-1,5-己二烯也被识别为聚苯乙烯的典型热解产物,判断其为苯乙烯类材料^[21]^。UVL图像表明,这些材料仅在剑柄附近的裂缝处发现,作为黏结剂使用。样品GBTJ-5、GBTJ-6的主要裂解产物为苯乙烯单体、苯乙烯二聚体和三聚体,表明该样品也是聚苯乙烯类材料。然而,它们的色谱峰形、应用部位和荧光颜色的差异暗示了两者之间的潜在差异。

**图9:**
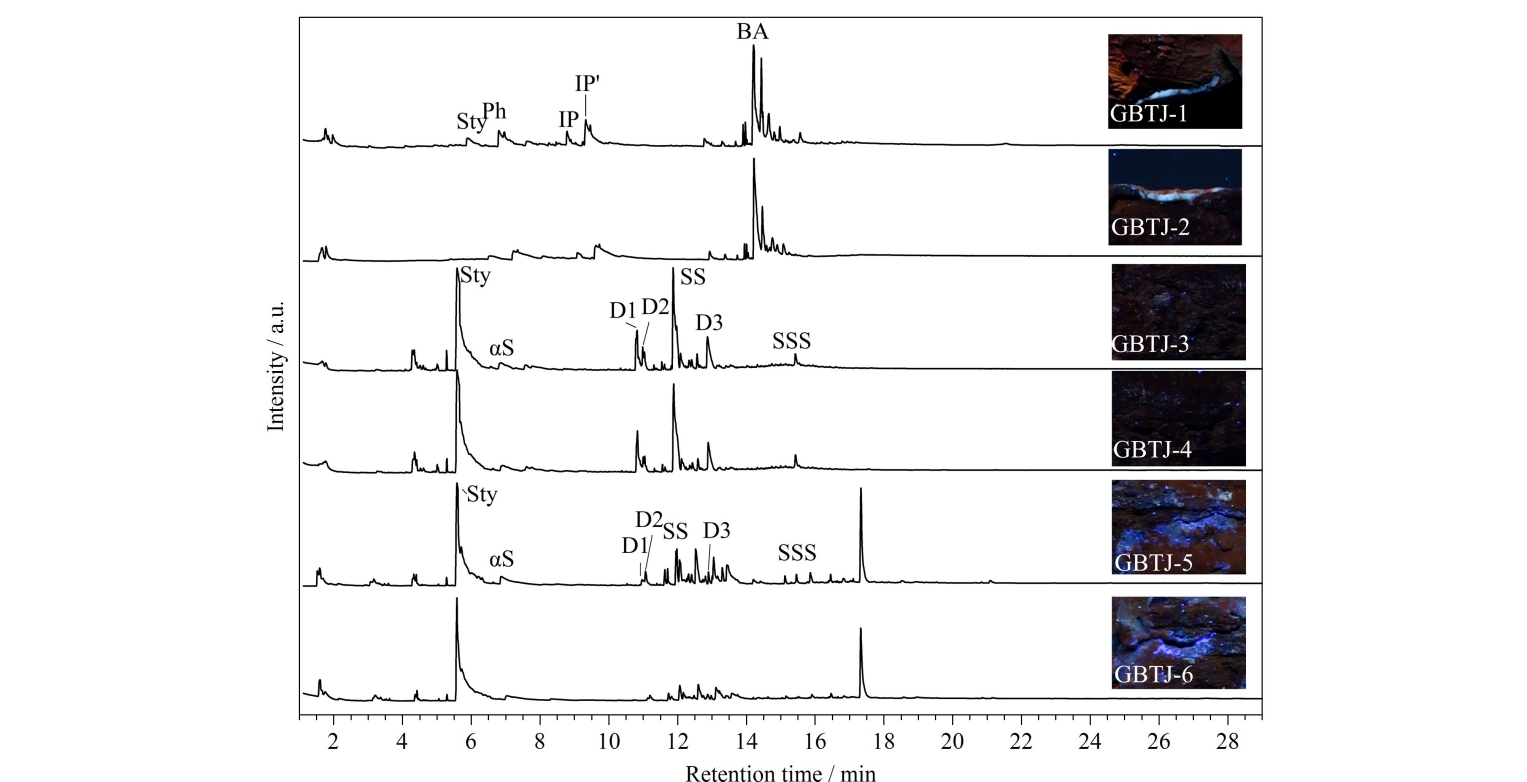
样品GBTJ-1~6的图像和总离子流图

样品GBTJ-7、GBTJ-8呈橙色荧光,样品GBTJ-9、GBTJ-10呈暗橙色荧光,热裂解分析结果(图10)显示这4个样品均含有虫胶的特征裂解产物,确认为虫胶。此外,样品中同时检测出苯乙烯单体,进一步证实了苯乙烯类材料在铁剑表面广泛分布。根据UVL图像显示的材料分布情况,虫胶分别用作黏结剂和调和颜料做旧,因虫胶中矿物颜料的含量不同,显示出不同的荧光颜色。

**图10:**
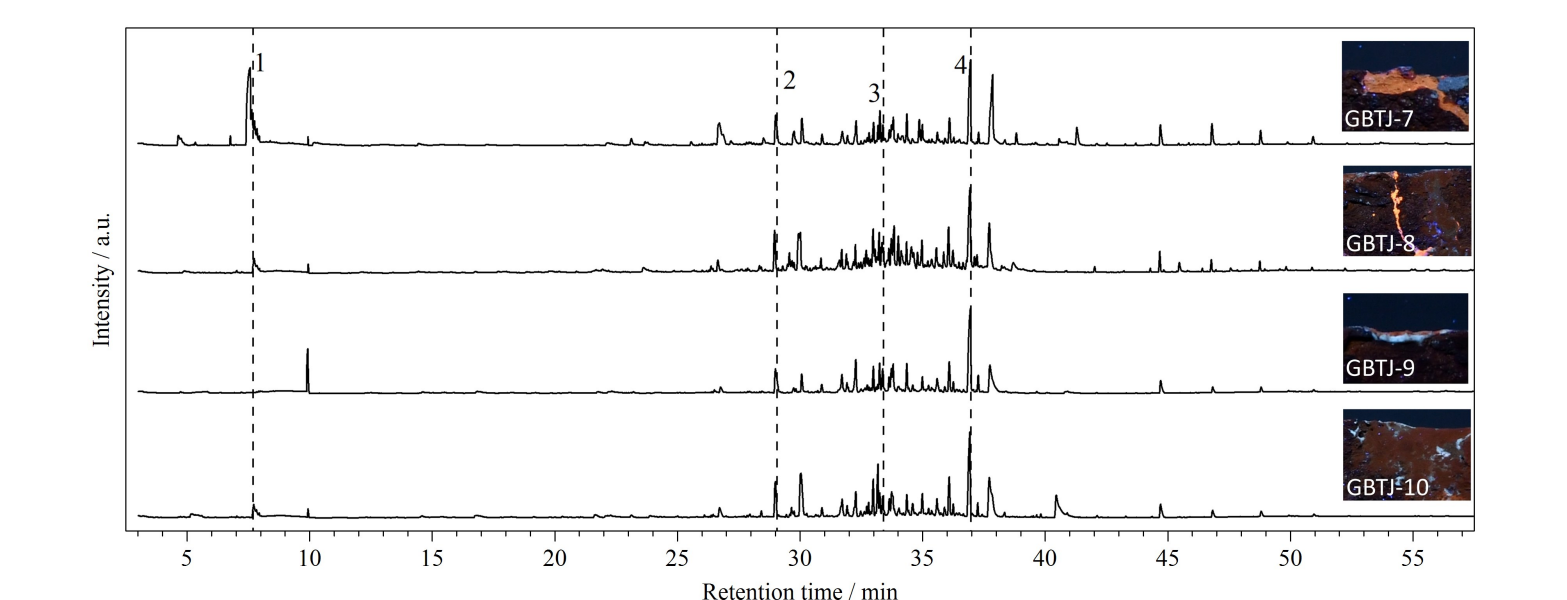
样品GBTJ-7~10的图像和总离子流图

综合以上分析结果,该铁剑修复过程中采用了双酚A型环氧树脂类作为粘接补配材料,剑柄附近的裂隙使用苯乙烯类材料粘接或渗透加固。此外,虫胶被用于粘接和调和色粉随色作旧。聚苯乙烯类材料作为封护剂,在铁剑表面广泛存在。

分析结果反映了修复师在修复材料选择上的多样性,根据文物的具体需求,灵活地运用了传统天然材料与现代合成高分子材料。自上次修复以来,铁剑450的保存状况稳定,结构完整无损,未发现新的腐蚀迹象,粘接和加固部分均保持良好,验证了20世纪70年代铁质文物清洗、除锈、脱盐、粘接和封护等保护方法的科学性和有效性。当前的检测结果为文物档案提供了重要补充。该铁剑在做好预防性保护的情况下,暂无需进一步干预。

## 3 结论

文物保护工作要求修复后的文物应整体协调,修复区域不易通过肉眼观察快速识别。UVL技术在调查铁质文物历史修复材料中展现了其优势,能有效揭示修复材料在文物表面的分布和保存状态。根据不同的发光颜色和强度初步判断修复材料异同,为取样策略的制定提供了重要依据,确保了样本的代表性,最大限度地减少了取样数量,减少了取样对文物的潜在损害。需要注意的是,材料的发光性质受到多种因素影响,如材料的种类和含量、老化降解情况、掺杂、多种材料的混合或叠压使用等都会影响其表面荧光性质。部分常用的保护修复材料如Paraloid B72,在紫外光激发下几乎没有发光(或特别微弱),因此没有发光现象并不代表没有使用修复材料。制定取样策略时,应综合考虑在可见光下的文物目视和显微观察结果。

通过对样品进行Py-GC/MS分析,在5件铁质文物上检测出熟桐油、虫胶、石蜡、环氧树脂、聚苯乙烯等多种保护修复材料。点分析和成像技术的结合揭示了材料的成分及其空间分布,获取了文物历史修复过程中有关材料的使用信息,为文物档案提供了重要补充,并为文物修复材料效果评估、失效保护材料去除以及保护方案制订提供了科学依据。
